# The Brief Case: Danger is afoot!

**DOI:** 10.1128/jcm.01693-25

**Published:** 2026-04-08

**Authors:** Eric J. Darrah, Macy Wood, Michael O. Frank, Rachael M. Liesman

**Affiliations:** 1 Department of Pathology and Laboratory Medicine, Medical College of Wisconsin 733705, Milwaukee, Wisconsin, USA; 2 Department of Medicine, Division of Infectious Diseases, Medical College of Wisconsin 196253, Milwaukee, Wisconsin, USA; Endeavor Health, Evanston, Illinois, USA

**Keywords:** mycetoma, aerobic actinomycetes, Acintomadura

## CASE

A 30-year-old man presented to the emergency department with left foot pain lasting several days, which worsened with weight bearing. On exam, a tender, dusky, soft tissue mass was observed on the dorsum of the left foot. The patient reported experiencing similar pain in the past, with multiple visits to local urgent care facilities. Intermittently, the lesion produced a white discharge. The patient was otherwise healthy and worked as a cleaner, which exacerbated his pain. Notably, the patient recalled a traumatic injury to this foot as a 12-year-old while residing in Mexico, from which he had immigrated 3 years prior. An MRI revealed a lobular mass involving the dorsal midfoot soft tissue and extending into the fourth metatarsal. An excisional biopsy was obtained, and tissue was submitted for histology and microbiology studies, including a Gram stain, aerobic and anaerobic bacterial cultures, fungal cultures, and mycobacterial cultures. Antibiotic therapy was deferred pending laboratory evaluation of the surgical biopsy.

**Fig 1:**
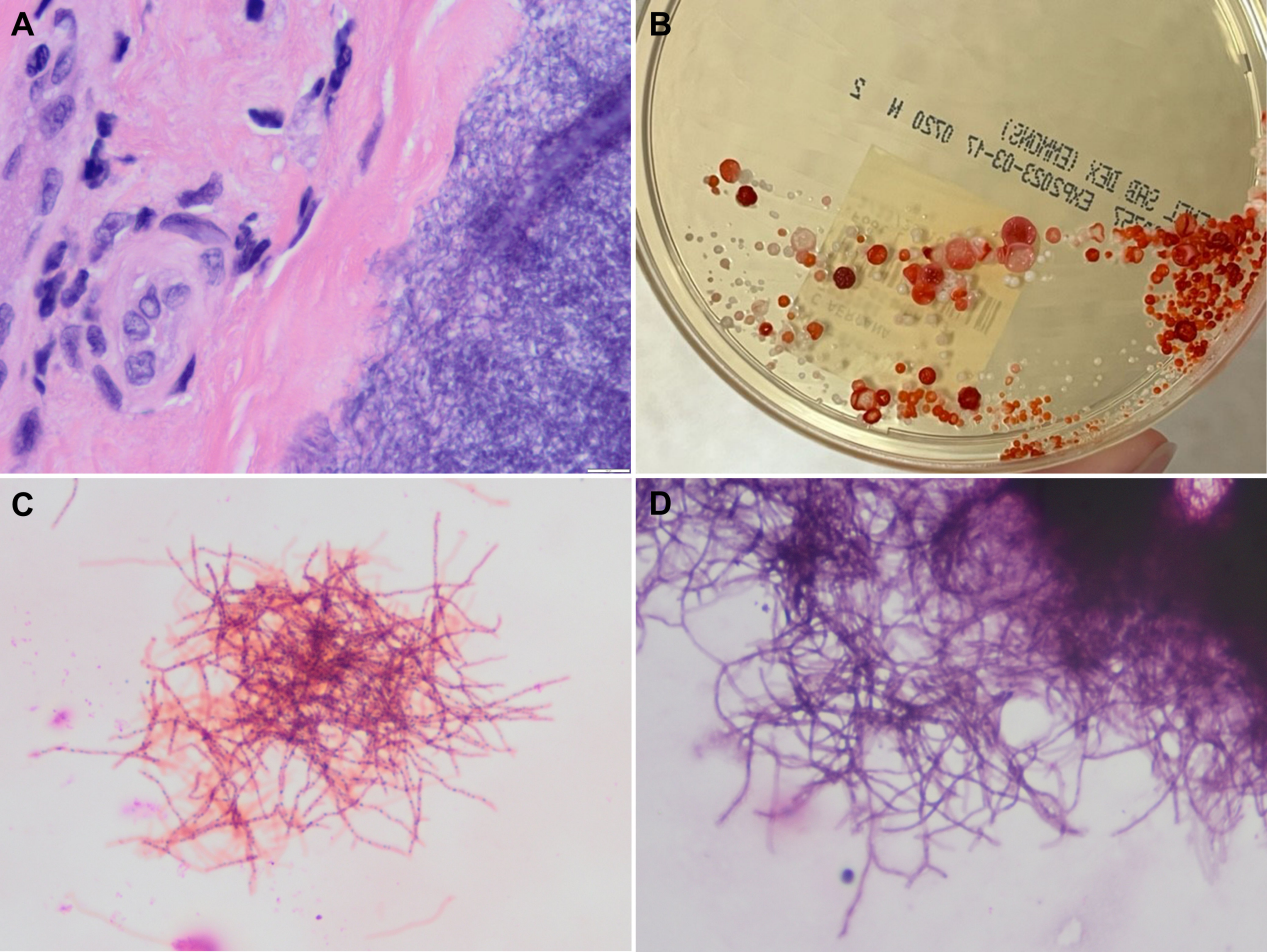
(**A**) H&E of the surgical biopsy specimen showing fibrosis and chronic inflammation adjacent to filamentous bacteria (40× magnification); (**B**) colony morphology on Sabouraud’s dextrose agar; (**C**) Gram stain of cultured colonies (100× magnification); (**D**) modified acid-fast stain of cultured colonies, interpreted as negative (100× magnification).

Branching Gram-positive rods, neutrophils, and red blood cells were reported from the Gram stain of the tissue. Histologic sections demonstrated a filamentous microorganism with mixed inflammatory infiltrate comprised primarily of plasma cells and histiocytes along with neutrophils (Fig. 1A). The microbes were positive by Gomori methenamine stain (GMS), positive by tissue Gram stain, and negative by Fite’s acid-fast stain. After 2 days of incubation, pinpoint white colonies were observed on blood agar from aerobic cultures, which were subcultured to Sabouraud’s dextrose agar for further workup. After further incubation, the colonies were orange-red and wrinkled in appearance (Fig. 1B). Gram stain of the colonies demonstrated filamentous, beaded, branching gram-positive rods, which were negative by modified acid-fast stain (Fig. 1C and D). Lysozyme testing was negative. Evaluation by matrix-assisted laser desorption/ionization time-of-flight mass spectrometry (MALDI-ToF-MS) did not match to a known reference spectra in the US Food and Drug Administration (FDA)-cleared or Research Use Only (RUO) library (Bruker Biotyper, MBT compass revision G 2023 library version). Colony morphology, negative modified acid-fast staining, microscopic morphology, and clinical features consistent with actinomycotic mycetoma were suggestive of *Actinomadura* species, but definitive identification required additional testing. The isolate was submitted for partial 16S rRNA gene amplification and sequencing, which identified *Actinomadura* species with 99.5% match to *Actinomadura madurae*. Susceptibility testing was performed by broth microdilution at a reference laboratory using Clinical and Laboratory Standards Institute (CLSI) M24S breakpoints for *Nocardia* and other aerobic actinomycetes (1). The isolate was susceptible to all antibiotics tested, including trimethoprim-sulfamethoxazole (MIC 0.064/1.190 µg/mL), doxycycline (MIC ≤ 0.12 µg/mL), amikacin (MIC ≤ 0.5 µg/mL), linezolid (MIC ≤ 0.5 µg/mL), ceftriaxone (MIC ≤ 1 µg/mL), and ciprofloxacin (MIC ≤ 0.12 µg/mL).

The patient was discharged with outpatient follow-up by infectious diseases and orthopedic surgery physicians. Management of actinomycotic mycetoma, also known as Madura foot, requires long-term antibiotics and surgical debridement, and a prolonged course of trimethoprim-sulfamethoxazole and doxycycline was planned. At 1-year follow-up, the patient described marked improvement to pain and function, although treatment had been complicated by intolerance to antibiotics resulting in periods of non-adherence to therapy.

## DISCUSSION

Mycetoma is a slowly progressive, chronic soft tissue infection, causing local tissue destruction. Patients generally present with a long-standing lesion following traumatic injury in regions of endemnicity (2). Often painful with palpable subcutaneous nodules, some lesions also produce serosanguineous discharge and may occasionally express macroscopic grains. Debilitating late-stage infections cause significant morbidity, with pain and local tissue destruction hindering form and function.

The majority of mycetoma cases are seen in tropical and subtropical regions where people walk barefoot. These regions include equatorial regions of Africa, Latin America, and Asia, colloquially the mycetoma belt, with the most cases reported from Mexico, Sudan, and India (2, 3). Greater than 75% of mycetoma cases occur in men, and ~70% of cases are associated with the foot, a syndrome sometimes referred to as Madura foot (3). Most cases are caused by aerobic actinomycetes (actinomycetoma), although mycetoma may also be associated with fungi (eumycetoma) (3). Aerobic actinomycetes are Gram-positive, soil-dwelling rods, with a worldwide distribution. Genera associated with mycetoma include *Nocardia*, *Streptomyces*, and *Actinomadura*. In Mexico, the majority of actinomycetoma is caused by *Nocardia brasiliensis* (78%) and *Actinomadura madurae* (9%) (4).

Histopathological examination of grains or excisional biopsy tissue is useful in diagnosis and differentiation between eumycetoma and actinomycetoma. Mixed suppurative and granulomatous inflammation is readily seen in hematoxylin and eosin (H&E) stain. GMS staining is useful for the visualization of fungal hyphae (eumycetoma) or thin bacterial filaments (actinomycetoma). If available, expressed grains should be examined by direct microscopy (5). Black grains are suggestive of eumycotic mycetoma caused by fungal organisms such as *Madurella mycetomatis*, *Trematosphaeria* (*Madurella*) *grisea*, and *Exophiala jeanselmei* (2). White or yellow grains are most commonly observed for actinomycetoma, although *Actinomadura* species have been associated with yellow-red grains (2, 6).

Culture and identification of the aerobic actinomycetes is challenging. The recognized diversity of aerobic actinomycetes causing human disease is broadening, and *Nocardia*, *Actinomadura*, *Gordonia*, *Rhodococcus*, *Tsukamurella*, *Streptomyces*, *Lawsonella*, and *Dermatophilus* are all recognized to cause human disease (6). Standard media (e.g., blood, chocolate, and Sabouraud dextrose agar) can be used for recovery of aerobic actinomycetes from sterile sites, while selective media (e.g., selective BCYE, modified Thayer-Martin) may be required to recover these organisms from non-sterile sites, such as respiratory samples (7). With the exception of *Lawsonella* species, all genera grow under aerobic conditions. Cultures should be incubated 2–3 weeks; routine bacterial culture incubation periods (5–7 days) are insufficient for recovery of aerobic actinomycetes. Therefore, clinical microbiology laboratories may elect to handle cultures of suspected aerobic actinomycetes with routine fungal cultures, ensuring adequate incubation and examination intervals. Aerobic actinomycetes may also be recovered from broth media, including mycobacterial cultures and routine blood culture bottles.

Microscopic features, including Gram stain morphology (e.g., branching rods versus cocci) and modified acid-fast staining characteristics, may facilitate preliminary reporting as an “aerobic actinomycete” or, for some select clinically relevant aerobic actinomycetes, a preliminary identification to the genus level. Modified acid-fast staining is most reliable when performed on direct specimens or mature colonies, as staining features may be absent or ambiguous before 7 days of culture growth (6). *Nocardia*, *Gordonia*, *Lawsonella*, *Tsukamurella*, and *Rhodococcus* stain modified acid-fast positive, while *Actinomadura*, *Streptomyces*, *Dermatophilus*, and *Dietzia* stain negative (Table 1). Additionally, morphologic microscopic features are useful in differentiating the most common clinically relevant aerobic actinomycetes (Table 1). *Actinomadura*, *Dermatophilus*, *Nocardia*, and *Streptomyces* demonstrate filamentous, branching morphology. In contrast, *Dietzia*, most *Gordonia* species, *Lawsonella*, *Rhodococcus*, and *Tsukamurella* have coccoid, coccobacillus, or rod morphology, and branching is rarely observed.

**TABLE 1:** Morphological characteristics of commonly identified aerobic actinomycetes^*a*^

Genus	Modified acid-fast	Microscopic morphology	Colony pigment	Colony morphology
*Actinomadura*	Negative	Thin, short, branching filaments	Blue, brown, cream, gray, green, pink, white, violet, or yellow	Wrinkled to leathery
*Dermatophilus*	Negative	Branching filaments, cocci (aerial hyphae)	Gray-white, yellow, orange	Rough, heaped colonies
*Dietzia*	Negative	Coccobacilli, rods	Yellow, orange, coral	Smooth
*Gordonia*	Positive (weak)	Short coryneform rods, cocci Some species exhibit branching	Beige, gray, cream, white, peach, orange, red, yellow	Smooth to slightly wrinkled
*Lawsonella*	Positive	Cocci, short rods	Colorless	Pinpoint, waxy
*Nocardia*	Positive (weak)	Thin branching filaments	Brown, pink, orange, red, purple, gray, yellow, peach, white	Chalky, matte, velvety, or wrinkled
*Rhodococcus*	Positive (weak)	Cocci, rods	Cream, yellow, orange, red, colorless	Rough, smooth, mucoid
*Streptomyces*	Negative	Branching filaments, short rods	White, yellow, or colorless	Lichenoid, leathery, creamy
*Tsukamurella*	Positive (weak)	Rods	White, orange	Creamy, small

^*a*^ Adapted from Brown-Elliott et al. (6).

Aerobic actinomycetes display a broad spectrum of colony morphologies with features overlapping across genera. Coloration spans the visible spectrum, and textures are variable and may shift as the colony ages (Table 1). While aerial hyphae are described in reference materials, cultures do not always produce these elements and may only do so after prolonged time or specific conditions, limiting their utility in diagnostic laboratories (5, 6).

Species-level identification of isolates is challenged by the number of pathogens and overlapping microscopic and morphologic characteristics. Excepting *Nocardia* species, proteomic identification by MALDI-ToF-MS is limited by the breadth and quantity of reference spectra in databases. Commercially available FDA-approved libraries demonstrate poor species-level identification of non-nocardial aerobic actinomycetes, which can be modestly enhanced with a custom library (8). Although manufacturers have expanded libraries in recent years, the majority of species remain only available in RUO databases. Currently, the FDA-approved Vitek MS (version 3.3) database includes 24 *Nocardia* species and *Gordonia bronchialis*, and the RUO database includes 28 *Nocardia* species and less than 10 species each for other aerobic actinomycetes, including *Gordonia*, *Streptomyces*, *Tsukamurella*, *Rhodococcus*, and *Dietzia*. The FDA-approved Bruker Biotyper CA database includes 5 *Nocardia* species and *G. bronchialis*, and the RUO database contains 89 *Nocardia*, 25 *Gordonia*, 165 *Streptomyces*, 11 *Tsukamurella*, 26 *Rhodococcus*, and 8 *Dietzia* species. Notably, neither FDA-approved nor RUO libraries for either manufacturer include spectra for *Actinomadura*.

Sequencing is currently the gold standard for identification of aerobic actinomycetes and has essentially replaced cumbersome and unreliable phenotypic methods (6). Sequencing of near full-length or full-length 16S rRNA gene (1,000–1,500 base pairs [bps]) is typically required to adequately differentiate closely related species. Previous studies demonstrated that the first 500 bp of the 16S rRNA gene are insufficient to reach this level of resolution (9, 10). Due to the relative stability of the 16S rRNA gene, a combination of 16S rRNA and alternate genes may be required to resolve closely related species, and several alternative sequencing targets have been investigated (6, 9, 11).

Treatment of actinomycetomas requires months to years of therapy, and recommended treatment regimens generally include trimethoprim-sulfamethoxazole and a second agent for pronounced or widespread infections (2). Antimicrobial susceptibility testing should be performed on clinically significant aerobic actinomycetes to guide appropriate therapy. Broth microdilution is the recommended method, and interpretive breakpoints are published in the CLSI M24S document, although application of these breakpoints to non-nocardial species is considered tentative (1, 12).

Aerobic actinomycetes are increasingly encountered in the clinical microbiology laboratory. While isolates are readily cultured on common media and *Nocardia* species have been incorporated into MALDI-ToF-MS libraries, non-nocardial aerobic actinomycetes generally require sequencing for complete identification. Clinical laboratorians must recognize aerobic actinomycetes, contextualize preliminary identifying information such as modified acid-fast and Gram staining morphology to clinicians, and establish procedures (in house or at a reference laboratory) for molecular-based identification and susceptibility testing of clinically significant isolates.

## SELF-ASSESSMENT QUESTIONS

1. Actinomycetoma may be caused by which of the following organisms?
  - *Actinomadura* species
  - *Exophiala* species
  - *Madurella* species
  - *Lawsonella* species
2. *Actinomadura* species can be best identified by which method?
  - 16S rRNA gene sequencing
  - Macroscopic morphology
  - Microscopic morphology
  - MALDI-ToF-MS
3. Typical treatment for actinomycotic mycetoma includes which of the following antimicrobial agents?
  - Amikacin
  - Penicillin
  - Streptomycin
  - Trimethoprim-sulfamethoxazole

## ANSWERS TO SELF-ASSESSMENT QUESTIONS

1. Actinomycetoma may be caused by which of the following organisms?
  - *Actinomadura* species
  - *Exophiala* species
  - *Madurella* species
  - *Lawsonella* species

Answer: a. *Actinomadura* species are common causes of actinomycetoma. *Madurella* and *Exophiala* species are common causes of eumycetoma. *Lawsonella* species are not associated with mycetoma.

- *Actinomadura* species can be best identified by which method?
  - 16S rRNA gene sequencing
  - Macroscopic morphology
  - Microscopic morphology
  - MALDI-ToF-MS

Answer: a. Sequencing is the gold standard for identification of aerobic actinomycetes. At the current state, the available commercial MALDI-ToF-MS databases are unable to identify *Actinomadura* species due to lack of spectra. Macroscopic and microscopic morphology may provide a presumptive identification, but these features overlap between genera.

- Typical treatment for actinomycotic mycetoma includes which of the following antimicrobial agents?
  - Amikacin
  - Penicillin
  - Streptomycin
  - Trimethoprim-sulfamethoxazole

Answer: d. Standard therapy includes sulfonamides (trimethoprim-sulfamethoxazole). Treatment duration is months to years.

**TAKE-HOME POINTS**

- Mycetoma is a slowly progressive subcutaneous infection, most commonly associated with traumatic injury of the foot in individuals residing within the mycetoma belt.
- Aerobic actinomycetes are the most common causes of mycetoma and grow readily on common media used in the microbiology laboratory, provided cultures are incubated for 2–3 weeks.
- Gram stain morphology and modified acid-fast characteristics of suspected aerobic actinomycetes are useful for genus-level presumptive identification but inadequate for species identification.
- Genus and species identification of aerobic actinomycetes remains a challenge in the clinical microbiology laboratory and, while MALDI-ToF-MS provides accurate identification of limited numbers of aerobic actinomycetes, sequencing is often required for complete identification.
